# The “business” of dentistry: Consumers’ (patients’) criteria in the selection and evaluation of dental services

**DOI:** 10.1371/journal.pone.0253517

**Published:** 2021-08-06

**Authors:** Laura Gray, Lisa McNeill, Weiming Yi, Anastasia Zvonereva, Paul Brunton, Li Mei

**Affiliations:** 1 Faculty of Dentistry, Department of Oral Sciences, Sir John Walsh Research Institute, University of Otago, Dunedin, New Zealand; 2 Otago Business School, University of Otago, Dunedin, New Zealand; 3 Division of Health Sciences, University of Otago, Dunedin, New Zeland; Danube Private University, AUSTRIA

## Abstract

The dimensions of patient-centred care include not only clinical effectiveness and patient safety, but, importantly, the preferences of patients as consumers of healthcare services. A total of 249 participants were included in the study, with a balanced population proportional representation by age, gender, ethnicity and geographic region of New Zealand. An online questionnaire was used to identify participants’ decision-making process, and what factors and barriers for participants to seek dental treatment. Cross-tabulations, Spearman correlation analysis and Pearson Chi-Square analysis were used for the statistical analyses. Three most common reasons for visit were check-up (77%), clean (57%) and relief of pain 36%). A desire to treat a perceived problem was the most common encouraging factor to seek dental care. Cost was the most common barrier to seeking dental services. The majority of participants attended a private practice (84%), with convenience of location and referral from professionals the most likely to influence their choice. Participants felt the most important trait a dental practitioner could demonstrate was to discuss treatment options with them before any treatment. Dental check-up, teeth cleaning and relief of pain were the most common reasons for patients to choose dental services. Cost and ethnicity of the consumers had a significant impact on how dental services were perceived and sought. Dental practitioners may need to reorientate how they express value of oral health practice, not just in regard to communication with patients, but also with government funding agencies.

## Introduction

Patient-centred care has become a universally recognised primary approach to high-quality health care amongst all health professionals. The dimensions of patient-centred care include not only clinical effectiveness and patient safety, but, importantly, the preferences of patients as consumers of healthcare services [1]. Previously, dental practitioners instructed and prescribed treatments with limited input from patients. Today, research studies recognise the importance of a patient-centred approach in the delivery of health care. Indeed, the governments, such as Australia, the UK, the US and the WHO, advocate and endorse the need for health care providers to place greater emphasis on the individual–the consumer of health services [2,3]. A consumer-driven style of healthcare delivery has been shown to generate significant benefits in patients’ physical and psychological outcomes, as well as in developing social health systems [3]. As a result, the consumer-driven approach has been adopted by many healthcare systems worldwide [4]. For example, the UK General Dental Council, in setting out the principles that dental professionals should follow, emphasised patients’ involvement in decision-making, their access and perceptions of value toward dental services [5].

Data from the New Zealand Oral Health Survey 2009 shows that poor uptake of dental services in New Zealand continues to be a concern. Low dental service attendance rates are common amongst men, young adults, Maori and Pacific Island New Zealanders, as well as those living in areas of higher deprivation [6]. It is known that good oral health is correlated to regular maintenance and professional assessment [7], however New Zealand consumer uptake of regular dental maintenance is still very low, particularly amongst some ethnic groups. For example, Maori adults in New Zealand are more likely to have tooth loss and untreated decay than non-Maori adults, and are almost two times as likely to have lost all of their natural teeth compared to non-Maori adults. The Maori population of New Zealand has the highest rate of decayed, missing or filled teeth in children, among all World Health Organisation (WHO) countries. This is a significant and enduring trend, and indicates a disconnect between dentist espoused service values and those perceived by these consumer groups [8].

Given this, the central question that most be asked is how do patients–the consumers of dental services–make their decisions? We–the providers of dental services–do not really know [1]. Although both theory (*i*.*e*. academic literature) and recommended practice (*e*.*g*. the NICE and NHS in the UK) endorse patient-centred care, the extent to which these dimensions have truly transferred into dentistry remains unknown [4]. Further, significant concerns remain regarding the impact of commercialisation and consumerism on the nature of oral health, and how dentistry services are provided [9]. For instance, there were a significant amount of grants ($25,738,621 according to the Work and Income New Zealand) provided for dental work in 2017, but half of NZ’s population were said to put off necessary dental treatment due to reasons such as cost [10]. It is thus crucial to investigate how patients (‘consumers’) make decisions regarding dental services; both for the benefit of patients, as well as the optimization of social health resources and development of dental practice.

The aims of the study were to identify what drives patients (‘consumers’) to seek dental services, what barriers to such services exist, and how perceptions of such service value may differ between key socio and demographic consumer groups. The objectives of the study were thus to survey New Zealand dental patients regarding their use of dental services. A key objective of the survey was to test the relevance of dental uptake motivational constructs, as identified in prior studies conducted outside of New Zealand. Further, the survey sought to attribute weighting to the factors influencing selection between dental providers, and pre- and post-service evaluations, within the study cohort.

## Methods

An online questionnaire was administered to 249 New Zealand residents (Table 1), with a balanced population proportional representation of participants by age, gender, ethnicity and geographic region of New Zealand. Participants over the age of 18 years, and fitting the demographic criteria outlined, were sought for inclusion in the study by Dynata, the only professional New Zealand market research company. Participants are contacted via email predominantly and are able to log into their panel home page at any time. Once within Dynata’s system, participants are matched with an available survey. An element of randomisation is part of the algorithm. Security checks and quality verifications are used on all sources before the participant can begin any survey. Additional quality measures include digital fingerprinting to prevent duplication, spot checking via third party verification to prove identity, reward redemption quality procedures, machine learning to identify and take action on unusual behavioural patterns, and benchmarking against known external data points. Dynata is able to deliver a sample that is nationally representative with the use of quotas and strict sampling plans in place. The survey was hosted on Qualtrics and ethics of the study were approved by the Ethics Committee of the University of Otago (D19/101). Informed consent was obtained from each participant.

**Table 1:** Participant demographics.

	Number (%)
**Gender**	
Male	120 (48%)
Female	129 (52%)
**Age**	
18–34	71 (28%)
35–44	61 (24%)
45–54	64 (26%)
55–64	53 (21%)
**Ethnicity**	
NZ European	164 (66%)
Asian	26 (10%)
Maori	33 (13%)
Pacific Islander	16 (7%)
Other	10 (4%)
**Income**	
<$20,000	23 (9%)
$20,000 - $39,999	36 (14%)
$40,000 - $59,999	40 (15%)
$60,000 - $79,000	40 (15%)
$80,000 - $99,000	36 (14%)
$100,000 - $149,000	61 (24%)
>$150,000	23 (9%)
**Region**	
North island	
Auckland	83 (33%)
Wellington	27 (11%)
Others	80 (32%)
South island	
Canterbury	33 (13%)
Dunedin	13 (5%)
Others	27 (11%)

**Fig 1:**
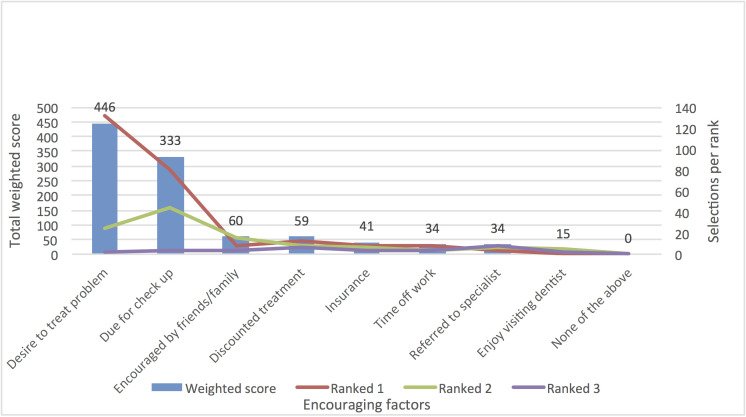
Drivers of dental service uptake.

**Fig 2:**
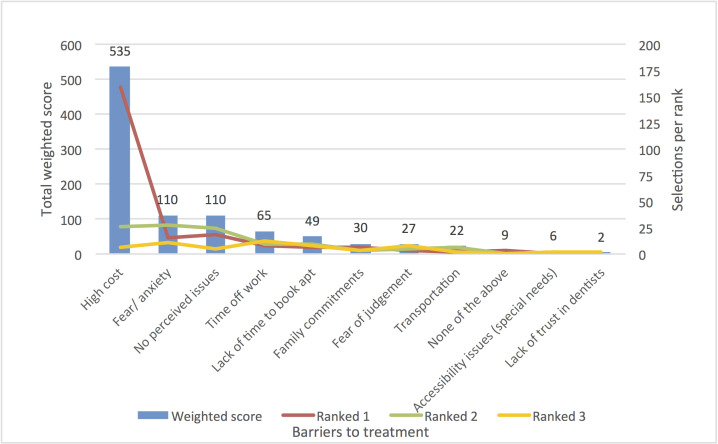
Barriers to seeking dental treatment.

**Fig 3:**
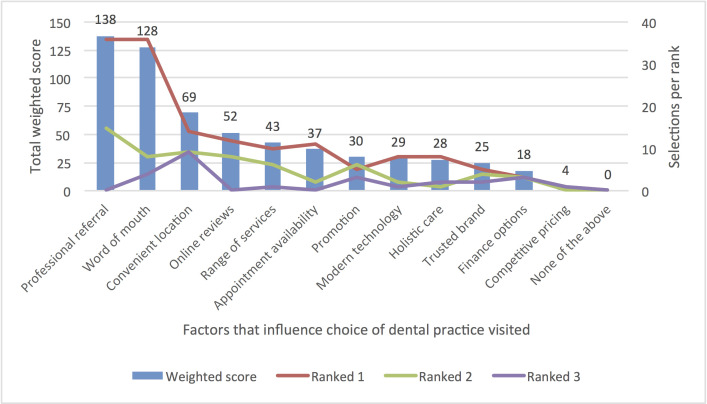
Factors influencing the choice of dental practice visited.

The questionnaire consisted of three main sections, the first determining dental patient treatment need, and asking participants to identify the reasons they had or had not sought a dental practitioner in the last two years. The second section of the survey examined nine factors that encouraged participants to seek dental treatment, as well as 11 barriers to seeking treatment (Figs 1 and 2). Participants were asked to identify factors in both instances, then rank their choices from most to least important in their final decision-making. The final section of the survey examined dental ‘practice’ factors, including what type of dental practice participants had attended, and the factors that influenced participants to choose this type of dental practice over others. In this section eight ‘practitioner’ factors and 13 ‘practice’ factors (Fig 3) were also investigated, with respondents asked to consider aspects of the practitioner (such as age and ethnicity) and practice (such as location and range of services) that influenced their choice. Responses were recorded via a 7-point Likert scale (1 = very important; 7 = not important at all).

### Statistical analysis

Data collected in the survey was analysed using SPSS (IBM SPSS Statistics, Version 23.0. Armonk, New York). Cross-tabulations were initially used to compare groups, with Spearman’s rank-order correlation used to measure the strength and direction of relationships between income, age, gender and treatment needs of participants. A Chi-square independence test was also used to examine associations between variables. Where participants were asked to identify and then rank key selections criteria, a weighted score was calculated for each factor. Weighted factor scores were cross-tabulated against demographic data, with one-way ANOVA used to test for relationships between demographic markers and individual selection factors.

## Results

### Motivations for dental service uptake

Overall, the most common reasons for considering dental services among participants were: check-up (77%), clean (58%), relief of pain (36%), critical care (31%), aesthetic concerns (15%), improving mastication (12%) and bad breath (8%). Of those study participants who reported visiting a dental practice for aesthetic concerns, 74% wanted to change the colour of their teeth, 31% wanted to change the alignment of their teeth, 41% wanted to change the shape or size of their teeth and 59% wanted to improve the appearance of missing or broken teeth. NZ Europeans were less likely to be concerned with aesthetics (χ^2^(1) = 4.002, *p = 0*.*045*) than were non-NZ Europeans in the study. Aesthetic concern decreased overall with increasing age (r_s_ = -1.81, *p = 0*.*003*).

Consistent with the motivations toward dental service consideration, the two most common drivers of actual engagement in dental treatment among all participants were a desire to treat a recognised problem, and being perceived as ‘due’ for a check-up (Fig 1). These two factors were also weighted as having the greatest influence on decision-making of all factors. When individual consumer segments were examined, participants with the highest incomes were more likely to consider a check-up (r_s_ = 0.143, *p = 0*.*021*) than were lower income groups. A higher income (χ^2^(33) = 47.804, *p = 0*.*046*) combined together with NZ European ethnicity (χ^2^(3) = 14.582, *p = 0*.*002*; F [3, 125] = 5.310, *p = 0*.*002*) was the consumer segment most likely to be motivated by being due for a check-up when considering dental care.

### Barriers to uptake of dental services

In regard to barriers to engaging in dental services, cost was overwhelmingly chosen as the key factor likely to prevent a participant from seeking dental treatment, followed by fear or anxiety regarding the dental service sought, and a lack of perceived current dental issues (Fig 2).

### Choosing between dental providers

The majority of participants in this study attended a general (all service) dental practice (84%) when seeking dental care. Choice of dental practice was influenced by convenience of location and word-of-mouth reputation of practice, followed by appointment availability. However, when ranked, a referral from another health professional was the most influential factor in the decision of which dental practice to attend (Fig 3). The referral factor had a significant impact on the decision of the type of dental practice chosen to visit across all participant ethnic groups in this study (χ^2^(6) = 44.053, *p < 0*.*001*). Gender and appearance of the dentist, promotions, appointment availability, range of services and modern technologies were less important factors to the participants of this study.

### Pre and post-service evaluations

Following the findings of prior research, participants were asked to indicate the level to which pre and post-service evaluation factors influenced their dental service decision making. Study participates did not consider ethnicity, age or gender to be an influential factor in selecting a dentist. The professional qualifications of the dentist did, however, influence choice (Table 2). Males particularly did not consider the age (F[6, 252] = 4.823, *p < 0*.*001*) and gender (F [6, 252] = 3.154, *p = 0*.*005*) of the dentist to be highly influential in their decision making.

**Table 2:** Pre-service evaluations of dental practitioners.

N = 249	Mean	Std. Error	Std. Deviation
The dentist’s ethnicity strongly influences my decision when seeking out a dental practitioner.	5.08	0.127	2.037
The dentist’s age strongly influences my decision when seeking out a dental practitioner.	4.95	0.109	1.749
The dentist’s gender strongly influences my decision when seeking out a dental practitioner.	4.46	0.118	1.893
The dentist’s qualifications strongly influence my decision when seeking out a dental practitioner.	2.39	0.086	1.378

1 = very important; 7 = not important at all.

Post-service evaluations factors, including practitioners discussing treatment options with them before commencement of any treatment, and demonstration of practitioner knowledge and understanding of treatment needs were deemed important by participants, as were pain management and timeliness of the dentist (Table 3). Females put the least emphasis on the importance of a dentist discussing treatment options before commencing (F [5, 253] = 3.672, *p = 0*.*003*).

**Table 3:** Post-service evaluations of dental practitioners.

N = 249	Mean	Std. Error	Std. Deviation
It is very important to me that my dentist demonstrates their knowledge and understanding of my treatment needs.	1.69	0.067	1.073
It is very important to me for my dentist to discuss my treatment options before commencement of any treatment.	1.63	0.061	.981
It is very important to me that my dentist does not cause any pain during treatment.	2.09	0.073	1.171
It is very important to me that my dentist is on time.	2.26	0.066	1.063

1 = very important; 7 = not important at all.

## Discussion

Prior research has found that major factors that deter individuals from seeking dental services were the high cost of such services, a lack of time to undertake dental care, dental phobias, dental practices not being accessible, not knowing who reliable dentists were, and expectations that the problem would resolve itself without intervention [11]. This study parallels some of these findings, with participants citing the perceived high cost of treatment, dental phobias and a belief that they had no dental issues as core barriers to engagement with dental services. In New Zealand, the cost of dental care is widely accepted as a significant barrier to uptake [6]. Oral health care for adults is provided by private dental professionals on a user-pays basis in New Zealand, with some public funding of dental services for low income adults, special needs and medically compromised patients, incarcerated individuals and all those who incur dental injuries through accidents. However, much of the funding available is for critical care only, not for routine oral health maintenance. Where Maori and Pacific Island populations are overrepresented among low-income groups in New Zealand, these communities are largely only subsidised for treatment of dental problems, not prevention. This is significant in relation to the findings of this study, where Maori and Pacific Island participants generally only sought dental services for relief of pain or treatment of critical conditions.

Only approximately half of the population in New Zealand are said to regularly attend the dentist [6]. In this study, the participants who did engage with routine oral health check-up services tended to be those with higher incomes, which would support the notion that the cost of dental services is the primary barrier to dental service uptake in this country. However, in New Zealand, all dental care (with the exception of aesthetic dental procedures) is free for children under the age of 18 years. In this study, there was a decline in the aesthetic concern overall with increasing age. Aesthetic dental treatment, such as orthodontics, is more popular in relatively younger patients. Ostensibly, one might thus expect that access to free care would result in good oral health across the child and youth populations of New Zealand. These indicate that there is more than cost preventing uptake of dental services among some consumer groups. The finding of this study that Maori adults are unlikely to perceive that they require any oral health care until they perceive a critical problem is therefore an important finding in better understanding barriers to dental service uptake, and pathways to improving oral health amongst this group.

The most frequent factors that influence the choice of dental practice selected were convenience of location and word-of-mouth reputation [12,13]. Participants in New Zealand are likely to seek recommendations from their friends, family, and other health providers, which concurs with extant literature. However, this study found that consumers rate online reviews of dental services as a highly influential factor in the decision-making process. This finding may reflect changes to the way individuals consume health care services generally, supporting literature that notes a shift toward health care being treated as a consumer ‘product’ and evaluated in a similar fashion. This is a useful finding for dental practice, as it exemplifies the need for better understanding of digital media management by healthcare practices.

It is interesting to note that access to finance or credit options, and competitive pricing ranked least among influential factors once consumers had made the decision to undertake dental services. This underscores prior discussions of the commercialised model of dental practise, where the aesthetic smile is seen as a symbol of socio-economic prestige [9]. While this may seem to contradict the earlier finding, that cost is one of the greatest barriers to uptake, this finding is consistent with the indication that the majority of consumers in New Zealand seek dental care for critical incidents and pain management. In this circumstance, quality of care and access to an appointment would likely be of more concern than overall cost.

Previous research has found that when deciding on a dental practitioner, patients have traditionally sought recommendations from their friends, family, the internet, other dentists and their insurance providers [14]. However, evidence suggests that dental service choice is neither clearly understood nor systematically assessed in most dental service provider settings [1,5]. Some research has been conducted exploring dentist related attributes in patient choice, with those attributes assumed to be of importance to most consumers are; quality of care, the number of years a dentist has been practicing, reputation, appearance, interpersonal skills, waiting times, technology offered and explanation of treatment/patient participation in treatment decision [12,14]. These factors are said to differ in importance depending on the characteristics of the consumer, such as their age and whether they sought comprehensive treatment or relief from pain [12,15]. However, the majority of existing dental choice research is US based, and hence applied in a setting that has a significant dental insurance provider impact on patient choice, with limited free decision making within the system. In a mostly private dental system, such as that of New Zealand, where dental insurance is minimal, it would be with some caution that these results could be assumed to apply.

To understand fully why consumers choose their dental practitioner, it is firstly important to consider that, normally, a consumer has already identified a problem that they believe needs to be addressed [16]. The form of this perceived need thus influences what a consumer will look for when choosing a dentist, and thus confounds attempts to qualify exactly what a consumer looks for in a dental service [12]. Where the literature does agree is in regard to the importance of a dentist’s interpersonal skills [17]. Early studies of dental service provider choice indicated courtesy, competence, reputation, and overall interpersonal skills as most important in evaluating a dentist [13]. However, assessment of interpersonal skills before the service decision is made is difficult. Recent studies, seeking to examine pre-selection perception criterion show that factors such as perceived competence of the dentist, recommendations from a friend, and the overall perceived quality of the dental service are used as proxy evaluations of the likely service outcome by consumers [17].

Evaluating the quality of a dental service before experiencing treatment is complex, forcing consumers to identify a set of key service quality indicators that they believe are inherent within dental practice. It has been found that the option to see the same dentist for each appointment, and how modern and well-kept the practice was, indicated likely experience quality [18]. However, the same study also indicated the after service factor of dentist sensitivity to concerns as the most important factor in dental practice choice, and the differences in the way men and women evaluated dental services, with women ranking factors such as the friendliness of the dentist high, and men ranking the practice facility appearance as more important.

Literature warns of the perils of treating dentistry as a commodity [4]. When selecting between dental service alternatives, clinical factors, such as treatment options available or technology access are said to be of concern to many consumers, with breadth of range of services offered by individual practices used as a proxy evaluation criteria for this prior to experience. This is interesting, as one might expect that expertise of the dentist is also indicated by specialisation, however the global trend toward conservation of natural dentition, rather than extraction, would explain a preference by consumers for a dentist who can offer multiple services. In New Zealand, evidence supports this claim, with a rapidly declining rate of tooth extraction–from 50% nationally in 1950 to just 9.4% nationally in 2009 [6,19].

Agreement on other factors of importance identified in the literature are mixed, with some studies citing quality of work, personal appearance of the dentist, waiting times and convenient location as drivers of dental service choice [14]. As these studies are based in insurance driven markets, the identified factors are likely to be influenced by the limited choice set faced by consumers, whose health insurance packages determine much of the initial treatment provider selection [18]. This explains the limited consideration of price as a core selection factor in the majority of literature (which tends to consider the US consumer market). When service price is explicitly considered, while a significant number of consumers agree that they consider price in their decision making (54%), only a small number cite price as a high priority amongst other decision factors (8%) [13]. Researchers note that findings such as this are likely to be driven by the insurance market of the US, where consumers would only be expected to pay a ‘top up’ amount over and above what is covered under their health insurance plan when selecting a more expensive service provider [13]. As dental insurance is relatively rare in the New Zealand context, price could be assumed to have a much more prominent role in dental service decision-making than shown in extant theory.

Related to the issue of dental insurance impact on service decision making is the limited understanding in extant theory of what might prevent a consumer from seeking dental care (beyond not having health insurance). In New Zealand, where dental provision is largely private, and the majority of consumers do not have dental health care insurance, a significant number may make the choice not to seek dental treatment at all (only 51% of the population regularly attend the dentist, while 40.3% are episodic/critical care users only) [6]. The prevalence of dental cavities in New Zealand is high among affluent, Westernised countries, suggesting a consumer perception in this country that dental issues are minor, and likely to self-resolve [11].

## Conclusions

This study highlights a central problem in patient attitudes toward dental care, in that the primary motivating factor was critical need for such care (especially relief of pain). Management of good oral health and preventative services such as check-ups or professional cleaning of the teeth were not motivators for seeking dental care. The most significant barrier to seeking dental services within the study population was perceived cost of service, suggesting that an economic model prioritising maintenance of oral health over critical care is required, and the current funding of critical care only in adults may be misplaced in regard to maintaining a healthy oral standard among New Zealanders. In terms of perceptions of service value, participants in this study were concerned with after service factors, including the availability and sensitivity of dentists in responding to patient concerns, indicating the importance of developing positive patient perceptions if the goal of encouraging maintenance and preventative care is to be reached.
